# Research on Cooperative Perception of MUSVs in Complex Ocean Conditions

**DOI:** 10.3390/s21051657

**Published:** 2021-02-28

**Authors:** Lili Yin, Rubo Zhang, Hengwen Gu, Peng Li

**Affiliations:** 1 College of Software and Microelectronics, Harbin University of Science and Technology, Harbin 150040, China; printing3d@hrbust.edu.cn; 2 Department of Computer Science and Engineering, Dalian Nationalities University, Dalian 116600, China; zhangrubo@dlnu.edu.cn; 3 703th Research Institute, China Shipbuilding Industry Corporation, Harbin 150080, China; guhengwen234@126.com

**Keywords:** multiple USVs, cooperative perception, complex ocean conditions

## Abstract

Since the working environment of Multiple Unmanned Surface Vehicles (MUSVs) is accompanied by a large number of uncertainties and various hazards, in order to ensure the collision avoidance capability of MUSVs in complex marine environments, the perception of complex marine environments by MUSVs is the first problem that needs to be solved. A cooperative perception framework with uncertain event detection, cooperative collision avoidance pattern recognition and environmental ontology model is proposed to realize the cooperative perception process of MUSVs using ontology and Bayesian network theory. The cooperative perception approach was validated by simulating experiments. Results show the effectiveness of cooperative perception approach.

## 1. Introduction

Compared with the research and development of Unmanned Aerial Vehicles (UAV) and Unmanned Ground Unmanned (UGV), Unmanned Surface Vehicles (USV) started late, but developed rapidly. In addition to a wide range of military applications, USV can be used in civilian applications such as environmental surveillance, search and rescue, navigation, and hydrographic surveys. The U.S. Department of Defense’s 2011–2034 Unmanned Systems Integrated Roadmap also identifies USV with autonomous navigation capabilities as an important research target [1]. However, a single USV is limited by the limited payload it can carry in the face of diverse missions. The cooperative system composed of MUSVs, which has more robustness, mobility, flexibility, higher operational efficiency, and wider operational range, has become a new form of USV application and has received widespread attention [2]. The US Navy’s “Master Unmanned Vessel The US Navy’s “Unmanned Boat Master Program” envisions a “high-return” mission scenario for USV, and more and more heterogeneous USV with different functions will be created. MUSVs can cooperate with UAVs, MUSVs, UGAs, etc. to form a combat system with strong environmental awareness and multi-dimensional spatial information acquisition capability.

However, the marine environment in which USV work is accompanied by a large number of uncertainties and various hazards, and factors such as currents, waves, tides, internal waves, storm surges, and turbulence can change depending on the working sea area, and these environmental factors are difficult to be predicted and accurately described [3].The high speed motion characteristics of USV and the complexity of the working environment bring some challenges to the research and design of MUSVs and practical applications of MUSVs pose some challenges. Therefore, in order to ensure the collision avoidance capability of MUSVs in complex marine environments, the sensing of complex marine environments by MUSVs is the first problem that needs to be solved.

This paper focuses on the cooperative perception technology of MUSVs in complex sea conditions, expecting to make a breakthrough in the theory and technology of the cooperative MUSVs, and to lay the theoretical foundation and technical support for the research of practical USV for safe and autonomous navigation in complex sea environments in China. Therefore, the research of this project has important theoretical significance and practical value.

In order to navigate safely and autonomously in the marine environment, a variety of sensors are used in the USV system to obtain environmental information. Such as GPS (Global Positioning System), ARPA (Automatic Radar Plotting Aid), AIS (Automatic Identification System), INS (Inertial Navigation System), optical and infrared vision systems, measuring sea winds, sea breeze, and sea breeze and other environmental sensors, and communication systems. The USV can use GPS and INS to obtain position information, use ARPA and AIS to identify dynamic targets and obstacles, and receive and process the targets. The USV can receive and process target and obstacle information, including its speed, heading, bearing, etc., during navigation [4]. USV can be considered as a mobile sensing node with multiple sensors, and the results obtained by each USV are local information relative to the marine environment and are redundant and complementary to each other. By collaborating with each other to sense the complex environment, we can share the sensing resources of USV, reduce the uncertainty and ambiguity of sensing, and improve the cooperative operation capability of MUSVs by sensing the movement intention and current working status of other USV. The environmental perception methods of unmanned systems at sea (including USV and MUSVs) mainly include analytical model-based methods, signal processing-based methods and information fusion methods [5,6]. Currently, the knowledge representation of USV sensing systems is mainly achieved by building perception models. As the amount of information in USV increases in the process of performing complex tasks, the use of ontological semantic knowledge framework to implement uncertain event models of USV has become a hot research topic. The ontology knowledge representation has good reusability and stability, and the reasoning capability of the ontology enables USV to process and understand the source data. For example, USV diagnostic systems using ontologies can locate possible faults based on the signs and warnings provided by the sensing system [7]. The ontology semantic knowledge framework approach not only improves the cognitive capabilities of the unmanned system, but also enhances flexibility, autonomy, and robustness in performing tasks [8]. The semantic knowledge framework proposed by E. Migueláñez et al. can detect and identify uncertain events such as task parameter changes and various sensor failures that occur in the task execution and system state of MUSVs [9]. The literature on cooperative perception of MUSVs is relatively scarce. The literature focuses on MUSVs for cooperative navigation and positioning at sea [10,11]. The research team relied on USV for the cooperative localization algorithm of MUSVs [12]. In the field of cooperative perception, a lot of research results have been obtained in mobile wireless sensor networks and mobile robots. The cooperative perception of MUSVs is mainly in the area of cooperative navigation and positioning, but the cooperative perception and information fusion of complex marine environments still need to be studied, especially in the representation of environmental models for knowledge sharing and information reuse, for which there is no clear implementation method yet.

To this end, the use of MUSVs to cooperative perception marine environmental information can improve the perception range and perception credibility. The specific research contents can be broken down as follows: (1) To facilitate information exchange among USV and reflect the sharing and reusability of knowledge, research on the knowledge representation and modeling methods of cooperative perception of MUSVs. (2) Research on the analysis and identification methods of uncertain events affecting the formation navigation of MUSVs. (3) Research on the determination methods of cooperative collision avoidance patterns of MUSVs that satisfy the international rules of collision avoidance at sea.

## 2. Cooperative Perception Architecture

**Figure 1:**
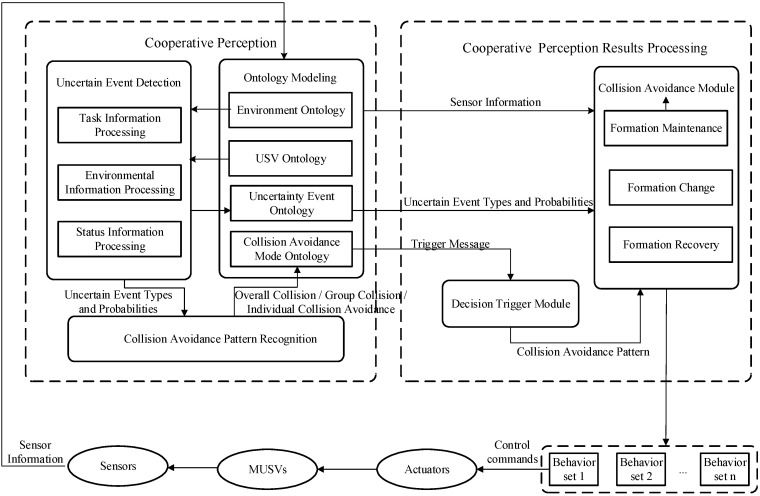
Cooperative perception process diagram.

Aiming at the background constraints of MUSVs performing formation safety navigation tasks in the marine environment and the flow characteristics of information during cooperative perception of MUSVs, the proposed architecture of cooperative perception includes [13]: uncertain event detection module, cooperative collision avoidance pattern recognition module, and environmental ontology model, as shown in Figure 1.

1. Uncertain events detection module: The possible obstacle-dense events, mobile vessel emergence events, course deviation events, key point unreachability events, deadlock events, etc. of MUSVs navigating in complex ocean environment will affect the safety of USV themselves. On the basis of fully summarizing and organizing uncertain events, we further analyze the causal relationship between uncertain events and formation navigation, and clarify the strategy for handling uncertain events, so as to provide a basis for USV formation navigation and cooperative collision avoidance planning. Firstly, the event state information from the uncertain event ontology is fuzzy quantified, and the quantified information is compared, analyzed and judged with the historical events and the event feature template in the domain, so as to calculate the probability of occurrence of the uncertain event.
2. Collision Avoidance Pattern Recognition Module: Extracts useful information from the raw data observed by the sensors and generates instances of certain concepts in the USV ontology, environment ontology, and uncertain event ontology. The new instances are then reasoned with rules and Abox to determine whether they are instances of key elements or events. If a key element or event instance is generated, the collision avoidance pattern processing module is triggered, and the OntoBayes method is used to apply Bayesian networks to the collision avoidance pattern ontology, and the degree of influence of uncertain events on the collision avoidance pattern is determined by using the Bayesian network inference algorithm to finally determine the collision avoidance pattern that MUSVs should take, and the identification results are stored in the collision avoidance pattern ontology.
3. Environmental ontology model: The purpose of environmental information ontology modeling for MUSVs is to represent the knowledge of environmental information for MUSVs using an ontology approach. Four ontologies can be established: USV ontology, environment ontology, uncertainty event ontology and collision avoidance pattern ontology. the USV ontology is used to describe the information of the USV platform itself, involving concepts such as position, attitude, velocity, direction of motion, other USV information and other USV system operating states; the environment ontology is used to describe the working environment in which the USV is located, and the main concepts are working area, key point, target point, obstruction, sea wind, waves, currents, visibility, etc. The uncertain event ontology is used to describe the uncertain event information that affects the formation of MUSVs, and involves concepts such as event name, event type, event probability, and event impact on each USV. In addition, the collision avoidance pattern ontology is used to describe the collision avoidance pattern that USVs should adopt according to the environmental perception results, including overall collision avoidance pattern, group collision avoidance pattern, and individual collision avoidance pattern, involving concepts such as pattern type, group number, and group member information.

## 3. Ontology Modeling of Environmental Information

MUSVs perform formation safety navigation tasks in complex ocean environments. Individual USV may also have different understandings and representations of uncertain events, which makes it difficult to share and reuse knowledge, making it more difficult to perceive the environment cooperatively. Ontology, as a modeling tool that can describe knowledge models at the semantic and knowledge levels, provides a normative description of concepts, lays the foundation for knowledge sharing and reuse, improves interoperability within and between systems, and uncovers some implicit data relationships. To this end, this paper applies the ontology to the modeling process of MUSVs environmental information ontology to realize its functions of representation, query, storage and processing at the semantic and knowledge levels [14].

However, so far, there is no ontology language that details a way to represent and reason about uncertain knowledge about concepts, properties, and instances inside a domain. This limitation of ontologies also limits the scope of ontology applications in the field of cooperative perception of MUSVs environments. Therefore, this paper probabilistically extends the ontology language OWL so that it can support the representation of uncertain information and combines Bayesian networks and ontologies to compensate for the deficiency of ontologies in uncertain reasoning.

**Figure 2:**
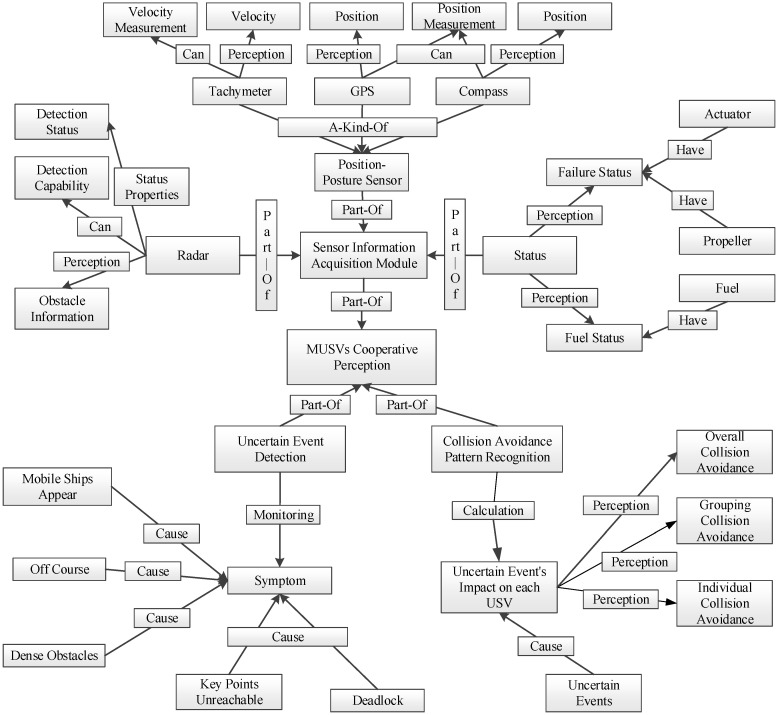
Semantic knowledge framework for cooperative perception application ontology.

Using the ontology approach to represent the knowledge of environmental information for MUSVs, four ontologies can be created: the USV ontology, the environment ontology, the uncertainty event ontology, and the collision avoidance pattern ontology. The semantic knowledge framework of the cooperative perception application ontology is shown in Figure 2.

The main concepts are: (1) Module: sensor information acquisition, uncertainty event detection, and collision avoidance pattern recognition; (2) Function: the ability of each type of sensor to process data on the environment, state, and task execution; (3) Sensor: all hardware included in cooperative perception; (4) State: the working health of each type of hardware in MUSVs; and (5) Information: the data to be cognized by the sensor.

The semantic knowledge framework of the cooperative perception application ontology builds semantic relationships among the nodes around the “MUSVs cooperative perception” node. The sensor information acquisition module, uncertainty event detection, and collision avoidance pattern recognition module are linked to the MUSVs cooperative perception system using Part-Of relationships; the sensor information acquisition is linked to the posture sensor and state awareness using Part-Of relationships; the posture sensor is linked to the tachometer, GPS, and compass using A-Kind-Of semantic relationships. The sensors and functions of MUSVs are attribute relations of Can; information and sensors are perceived and sensed relations. Through the ontological semantic knowledge framework, the information obtained by each sensor of MUSVs stimulates the cooperative perception process, and the cooperative perception motivates the decision and execution modules by establishing semantic relationships with MUSVs, which in turn enables reasoning capabilities similar to those of human minds.

**Figure 3:**
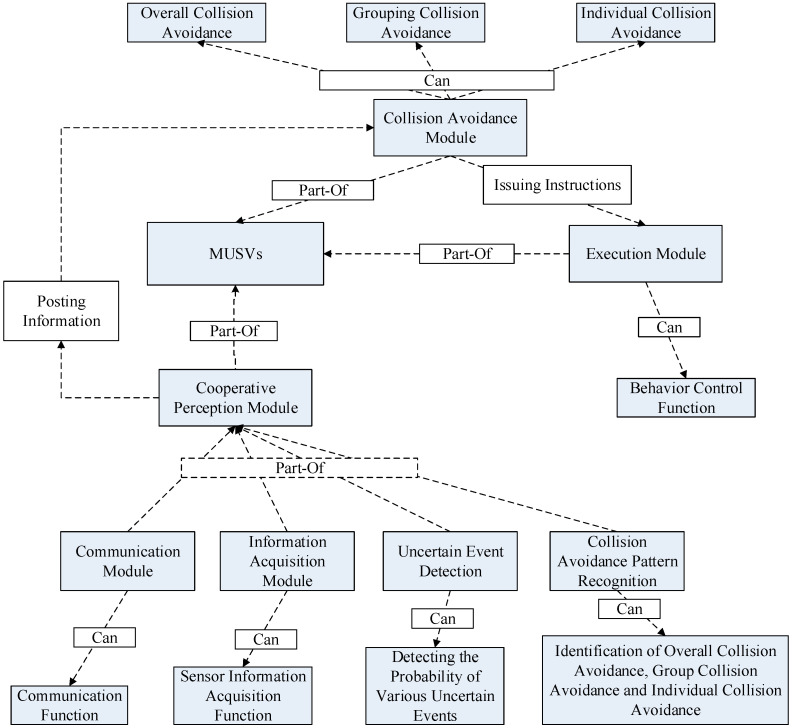
Ontology semantic knowledge framework of MUSVs formation safety navigation.

The logical relationship between the synergy module and the collision avoidance module when MUSVs perform the task of safe navigation in formation can be represented by an ontological semantic knowledge framework. The semantic knowledge framework expresses the synergistic process by means of a graphical representation. The formation safety navigation task ontology establishes the semantic knowledge framework of the cooperative perception, decision and execution modules through MUSVs to establish semantic relationships, as shown in Figure 3.

Figure 3 The semantic knowledge framework of the formation safety navigation task is constructed by the semantic knowledge framework fusion above, and the execution of the formation safety navigation task requires all modules of MUSVs to be realized cooperatively. Since the semantic knowledge framework of the cooperative perception modules is constructed in detail above, the semantic knowledge framework only needs to establish semantic relationships with important concepts and functions as nodes.

The various concepts included in the execution of formation safety navigation tasks by MUSVs can be described by the gray box diagram in the figure; the relationships between concepts can be described by the white box diagram; and the connections between concepts can be described by the dashed lines. The MUSVs in Figure 3 include the cooperative perception, collision avoidance, and execution modules. The cooperative perception, collision avoidance and execution modules are linked to the MUSVs using Part-Of semantic relations. The corresponding function of each module has an attribute relationship with that module. In addition, the collision avoidance and execution modules also establish attribute relationships with their own functions. With this semantic knowledge framework, the MUSVs can react quickly to uncertain events. For example, the cooperative perception module calculates that the occurrence of an event on the mobile vessel has a high degree of impact on the current USVs to complete the task of safe navigation in formation, and then calculates the impact of the uncertain event on each USV, and then identifies the overall collision avoidance, group collision avoidance, and individual collision avoidance, and outputs the collision avoidance pattern to the collision avoidance module, which makes a decision and gives a command to the execution module, and the execution module receives the command to After receiving the command, the execution module readjusts the angle and speed of the MUSVs.

## 4. MUSVs Cooperative Perception Method Based on Ontology and Bayesian Network Theory

### 4.1. Uncertain Event Detection

**Figure 4:**
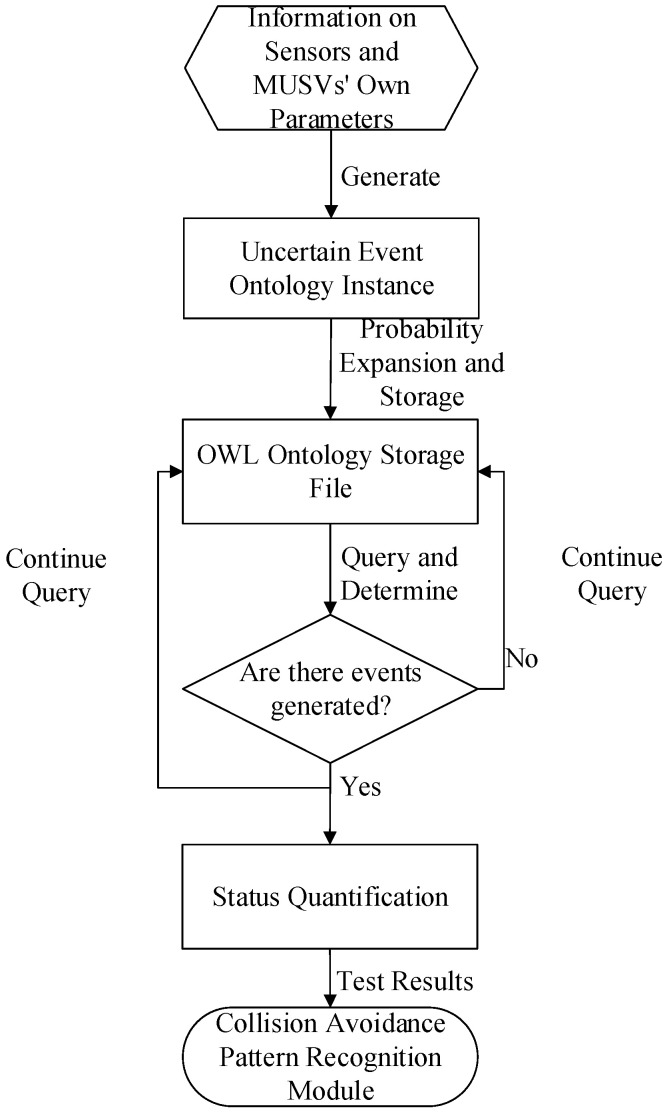
Flow chart of uncertainty event detection.

At the initial stage of MUSVs environment cooperative perception uncertain event detection, relevant information is first extracted from the raw data observed by sensors and used to generate instances of certain concepts well defined in MUSVs ontology, environment ontology, uncertain event ontology and collision avoidance ontology, i.e., to get the level of rising from raw data and information to semantic knowledge, with the goal of identifying the occurrence of relevant events from the underlying sensor data. The new instances generated are then reasoned using rule-based reasoning to determine whether they are instances of the relevant events. If the event occurs then the fuzzy logic event detection process is triggered. Fuzzy logic-based uncertain event detection fuzzifies the specific values of the event state information passed by the uncertain event ontology modeling, thus quantifying the event state. For different events, different model parameters can be selected for the establishment of the event state affiliation function. The quantified information is compared, analyzed and judged against historical events and the event pattern class feature template in the domain to calculate the probability of occurrence of the uncertain event. The event detection phase and will deposit the event type, event name and event occurrence probability as attributes of the uncertain event into the uncertain event ontology and trigger the collision avoidance pattern recognition process. The uncertain event detection process is shown in Figure 4.

(1) Uncertainty event detection rule design

The SWRL rules are constructed to identify MUSVs uncertain events, and some of them are shown in Table 1.

**Figure 5:**
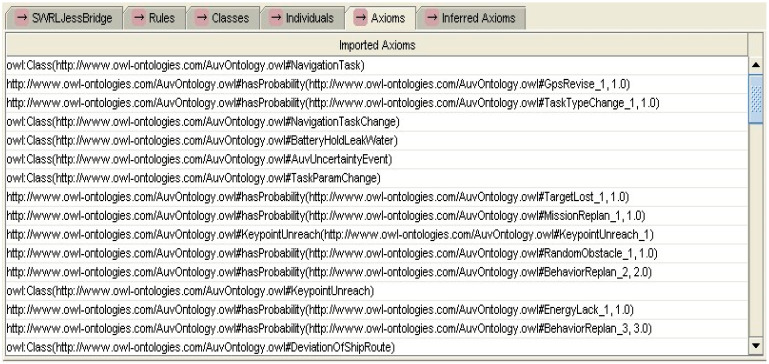
Experimental axioms for event detection inference based on SWRL rules.

**Figure 6:**
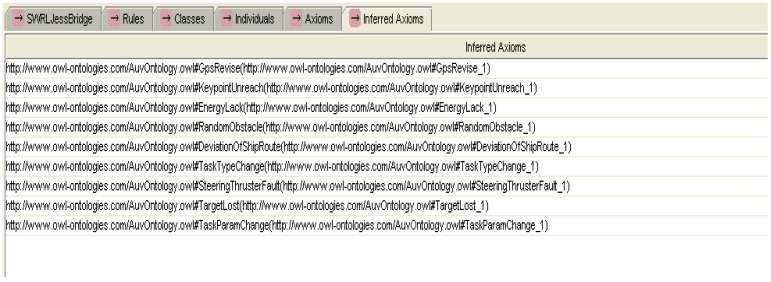
Experimental results of event detection inference based on SWRL rules.

In the cooperative perception process of MUSVs, if the various types of sensor information satisfy the SWRL rules for uncertain event detection, then the occurrence of uncertain events can be inferred from the specified axioms and rules. Figure 5 shows some of the experimental axioms for uncertain event detection inference based on SWRL rules. Figure 6 shows the experimental results of partial SWRL axiomatic reasoning in the cooperative perception process of MUSVs. From the experimental results, it can be seen that the SWRL rules for uncertain event detection of MUSVs developed in this paper can achieve effective results.

(2) Probability expansion of uncertain event ontology

**Figure 7:**
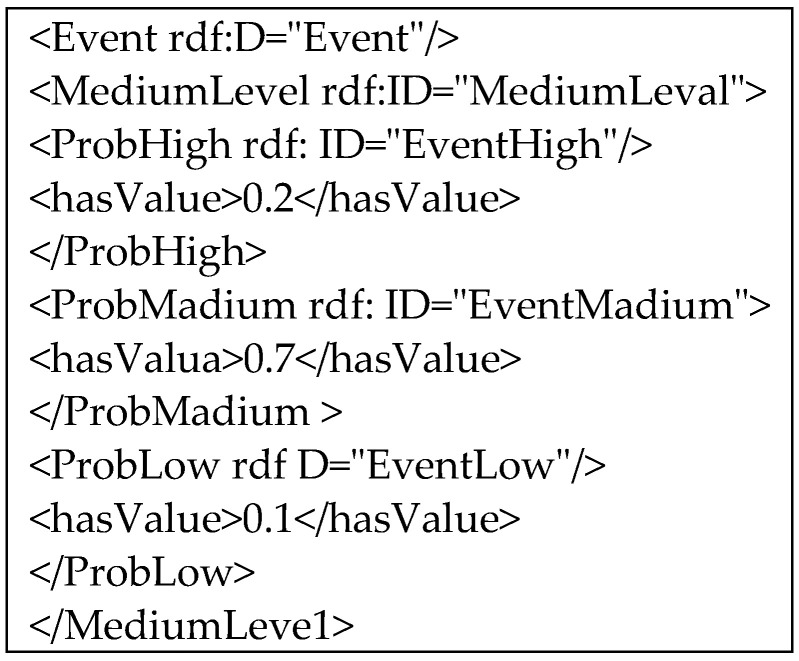
Instances of the medium rank uncertainty event.

In the process of uncertain event detection in MUSVs, the uncertainty knowledge between uncertain event classes and their attributes can be represented by probabilistic constraint relations, which in turn enables the function of ontology description probabilistic knowledge. Since the language cannot represent the uncertainty of uncertain event classes, this paper implements the probabilistic extension of uncertain event ontologies and collision avoidance ontologies by means of probabilistic extensions to the description language. Data type attributes are introduced; classes are introduced to represent the probabilities of classes and attribute nodes, which contain subclasses (low level probability), (medium level probability) and (high level probability). The above classes and attributes allow a more comprehensive description of the probabilistic knowledge of the cooperative perception process. For example, the instances of the medium rank uncertainty event, with respect to the ontology classes, and, are defined as, and, respectively, initialized with probabilities 0.2, 0.7, and 0.1. In a language with probabilistic extensions, this can be described as Figure 7.

The probabilistic information of uncertain events described in a language with probabilistic extensions is arranged in the order from high, medium and low. Due to the real-time requirements of the uncertain event detection process, the underlying ontology knowledge base also needs to be updated in real time.

The output of the MUSVs uncertain event detection is a file, which stores and updates in real time the identified uncertain event names, ranks and corresponding probability values, which are also used as input information for the collision avoidance pattern recognition module.

### 4.2. Collision Avoidance Pattern Recognition Method for MUSVs Based on Bayesian Network Theory

The collision avoidance pattern recognition module receives information such as uncertain event type, level and probability of occurrence of uncertain events from the uncertain event detection module. The relevant instances in the uncertain event ontology file are subjected to Bayesian network uncertainty inference to calculate the degree of impact of the current event on each USV and identify the collision avoidance pattern. Moreover, instantiate it into the collision avoidance ontology, which facilitates the MUSVs collision avoidance module to achieve fast decision making through the results of collision avoidance ontology query.

**Figure 8:**
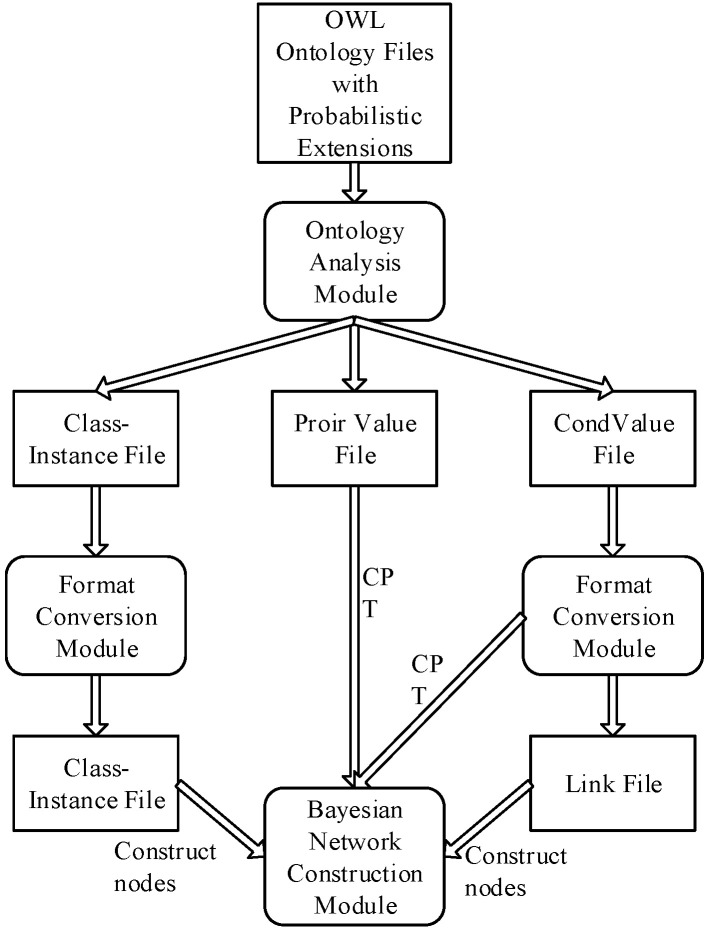
Overall framework of OntoBayes system.

For the characteristics of MUSVs performing tasks, a method is used to apply Bayesian networks to MUSVs collision avoidance ontology for uncertainty inference. First, a C program is written to parse the ontology file described in the language. The classes, conditional probability scores and prior probabilities in the ontology are saved in the, and files, respectively; then the C program is written to construct a Bayesian network according to the rules for constructing a Bayesian network; finally, the uncertainty event model and the constructed Bayesian network are used to perform collision avoidance patternl based on the real-time sensor information. The data flow is shown in Figure 8.

(1) Ontology Parser Design for Uncertain Event Ontology

The concepts and instances, prior probabilities and conditional probabilities in the ontology described by the language with probability extensions are parsed and saved in a txt file. After parsing, the files, and are generated to represent the class and instance relationships, prior probabilities and conditional probabilities in the MUSVs uncertain event ontology, respectively.

(2) Construction method of Bayesian network

The construction of Bayesian network mainly consists of three core steps: firstly, the Bayesian network nodes are generated, in which the class, the instances contained in the class and the prior probability information corresponding to the instances should be reflected; then the edges in the Bayesian network indicating the implied causality are generated based on the class-instance information; finally, the Bayesian network conditional probability table is generated, in which the conditional probability of the Bayesian network indicates the occurrence of the parent node under the condition that the child node The conditional probability of Bayesian network represents the probability of occurrence of child nodes under the condition that the parent node occurs, i.e., the influence of parent node on child nodes. The conditional probability table of the Bayesian network can be obtained from the prior probabilities output by the ontology resolution module.

**Figure 9:**
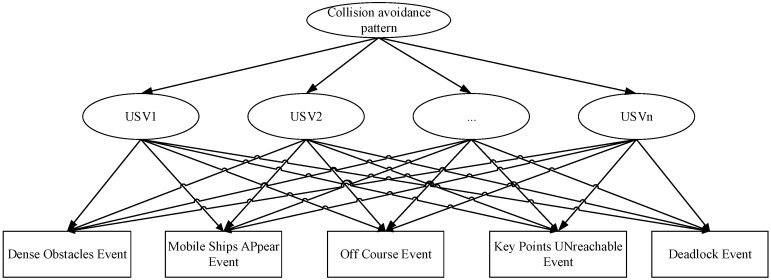
Bayesian network diagram of MUSVs collision avoidance pattern recognition.

The Bayesian network conditional probability table and the Bayesian network structure created by writing a C program according to the rules for establishing Bayesian networks, combined with the contents of the ClassInstance.txt, Condvalue.txt and Proirvalue.txt files are shown in Table 2 and Figure 9, respectively. The real-time input information of the Bayesian network includes not only sensor information, but also initialization information such as conditional probability and prior probability. p (ThreatLevel) = (0.3,0.4,0.3) indicates that the probability of MUSVs overall collision avoidance, group collision avoidance or individual collision avoidance is 0.3 for high, 0.4 for medium and 0.3 for low.

The inference process of the Bayesian network is: assuming the network structure of the Bayesian network model, the node X possesses a parent node U and m sub-nodes $Y_{1},\ldots ,Y_{m}$, which is defined as:

Bel: the reliability value of the node X, i.e., the posterior probability distribution;

λ The probability of diagnosis from subnodes, and the effect of the occurrence of result events on the reason for diagnosis. For leaf nodes, it is the probability of this probability event;

π Causal probability, which reflects the causal influence from the parent node and the brother node, is the prior probability for the root node.

Starting from the new event information or prior knowledge, the network obtains the causal probability from the parent node, obtains the diagnostic probability from the child node, updates its own reliability, and then spreads the effect of its own reliability update to other nodes:

In the first step, the reliability of this node is updated according to the newly obtained information, $Bel(x)=\alpha \lambda (x)\pi (x),\;\lambda (x)=\prod_{j}\lambda_{Y}(x),\;\pi (x)=\pi_{X}(u)\times M_{X|U}^{}$

The second step, the bottom-up propagation, $\lambda_{X}(x)=\lambda (x)\times M_{X|U}$.

The third step, updating from top to bottom, $\pi_{Yj}(x)=\alpha \pi (x)\prod_{k\neq j}\lambda_{y_{k}}(x)$, where $\pi_{X}(x)$ is the probability of causal prediction from node U to X, and $\lambda_{Y_{j}}(x)$ is the probability of event diagnosis from child node $Y_{j}$ to X. The normalization operator α guarantees $\underset{x}{\sum }BEL\left(x\right)=1$. The evaluation result given by the Bayesian algorithm is a probability vector corresponding to each state of the threatening node, viz $Bel(x)=P(x_{1},x_{2},\ldots ,x_{n})$.

MUSVs cooperative perception computes the collision avoidance pattern through a Bayesian network, where the child node is an uncertain event output by uncertain event detection and the parent node indicates the collision avoidance pattern. The MUSVs module can identify collision avoidance patterns by using Bayesian network uncertainty inference algorithm, combined with the conditional probability matrix of ontology analysis.

## 5. Navigation Task Cooperative Perception Simulation Experiment

The task scenario is designed for MUSVs to perform formation safety navigation, and uncertain events may occur during the navigation of MUSVs through a navigation area, which triggers the collision avoidance pattern identification process to determine whether the system should adopt the overall collision avoidance, group collision avoidance or individual collision avoidance pattern. The uncertain events that may occur in the task of safe navigation of a formation include uncertain events in the marine environment such as the appearance of moving vessels, unreachability of key points and course deviation [15].

**Figure 10:**
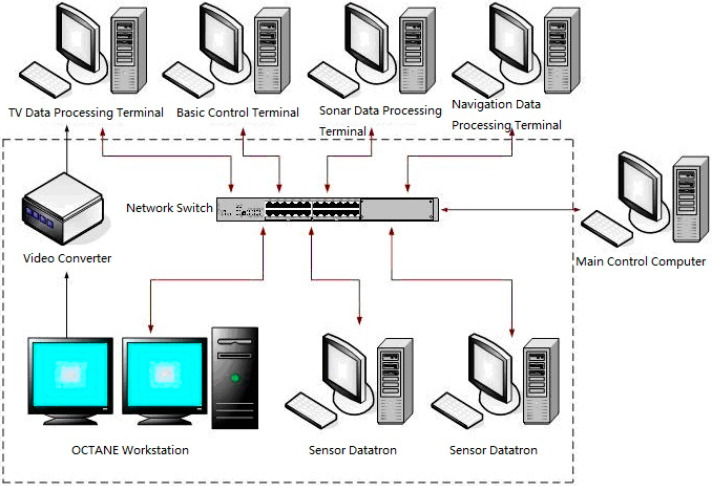
MUSVs integrated simulation system.

**Figure 11:**
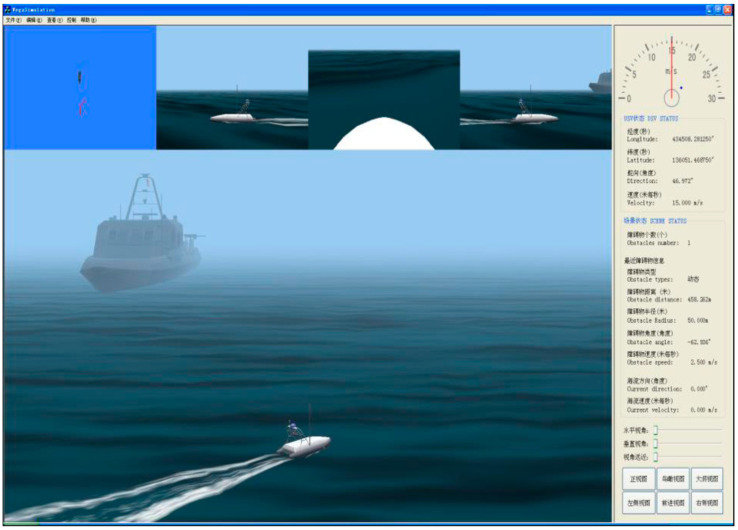
Interface of visual simulation.

**Figure 12:**
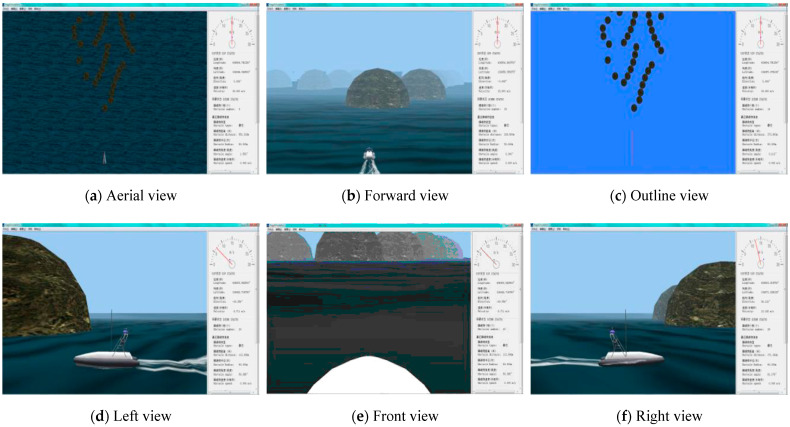
Views of graphic workstation.

MUSVs cooperative perception utilizes the MUSVs integrated simulation system built with SGI OCTANE2 workstation as the core. As shown in Figure 10, the components of the integrated simulation system are shown in the dashed box. The existing integrated simulation system can be used for the simulation of MUSVs by adding/changing functions. Interface of visual simulation and views of graphic workstation are shown in Figure 11 and Figure 12.

The experiment detects the probability of occurrence of marine environment uncertain events such as mobile ship emergence events, critical point unreachability events and course deviation events in the formation safety navigation task, identifies collision avoidance patterns, and updates the uncertain events and collision avoidance ontology knowledge base model in real time.

At the beginning of the simulation, no information such as obstacles and currents are added, no corresponding marine environment uncertain events are generated at this time, and no uncertain events are generated in the uncertain event ontology model, i.e., no corresponding OWL ontology instance content is generated in the underlying knowledge base, at which time the MUSVs navigate normally according to the set target.

(1) Mobile vessel emergence event

Mobile ship emergence is when MUSVs are on a mission and the distance between the MUSVs and the mobile ship is less than the safe distance, which will cause damage to the MUSVs if they do not evade in time. Initially, MUSVs sail towards the target point. After sailing for a certain distance a static obstacle is added at 100 places on the MUSVs’ sailing route with a radius of 40 m; when MUSVs and the obstacle are close to the process, a random obstacle emergence uncertainty event occurs with a certain probability. The cooperative perception results are shown in Table 3.

**Figure 13:**
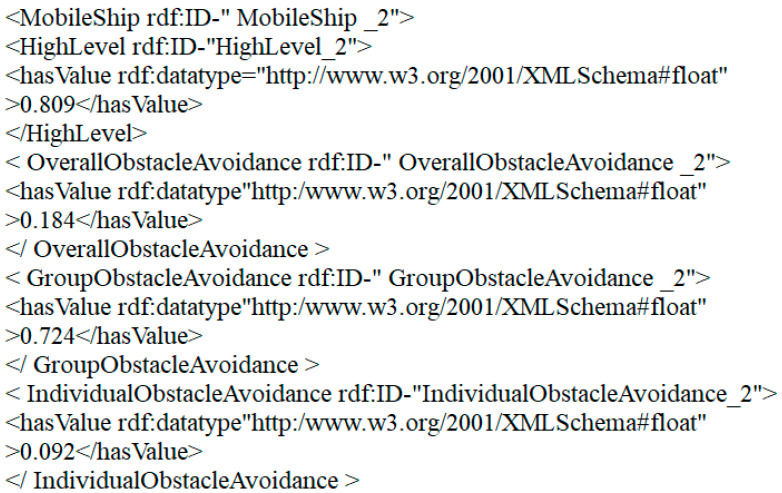
OWL format file.

The results of cooperative perception show that the system appears to require the MUSVs to invoke the collision avoidance procedure in a timely manner due to the proximity of the moving vessels to the MUSVs. At 100 s of system operation, the probability of occurrence of the mobile vessel emergence event is 0.723, and the grouped collision avoidance probability exceeds the trigger collision avoidance replanning threshold of 0.6. This result is reported to the environmental ontology modeling module, which generates the instance of the mobile vessel emergence event, the probability of occurrence of this event, and the collision avoidance pattern probability in the underlying knowledge base. At this time, according to the underlying rules, it is judged that the current uncertain event to group collision avoidance probability satisfies the conditions for triggering the collision avoidance module, and the system calls the collision avoidance program and then adjusts the speed and direction of MUSVs in time. The probability of the event occurring gradually decreases until the probability of the event occurring on the mobile ship is 0 at 107 s, and the overall collision avoidance probability, group collision avoidance probability and individual collision avoidance probability are restored to the initial values of probability 0.3, 0.4 and 0.3. Figure 13 gives the content of the corresponding OWL format file generated by the bottom layer when the event occurs on the mobile ship in the experiment.

Where MobileShip_2 represents the instance of the generated mobile ship occurrence event, HighLevel_2 represents its event level, here is high level, 0.809 indicates the probability of occurrence of this event. OverallObstacleAvoidance_2 overall avoidance, 0.184 indicates the probability of current overall avoidance. GroupObstacleAvoidance_2 group avoidance, 0.724 indicates the probability of current group avoidance. individualObstacleAvoidance _2 individual avoidance, 0.184 indicates the probability of current individual avoidance.

(2) Key point unreachable and moving ship emergence event occur simultaneously

Key point unreachability refers to MUSVs’ repeated attempts to reach the target during the mission due to the sub-target point of MUSVs’ navigation being occupied/encircled by obstacles, or invalid planning behavior, etc. The addition of two mobile vessels in close proximity during the navigation of MUSVs leads to the simultaneous occurrence of key point unreachability events and random obstacle presence events. The cooperative perception results are shown in Table 4.

**Figure 14:**
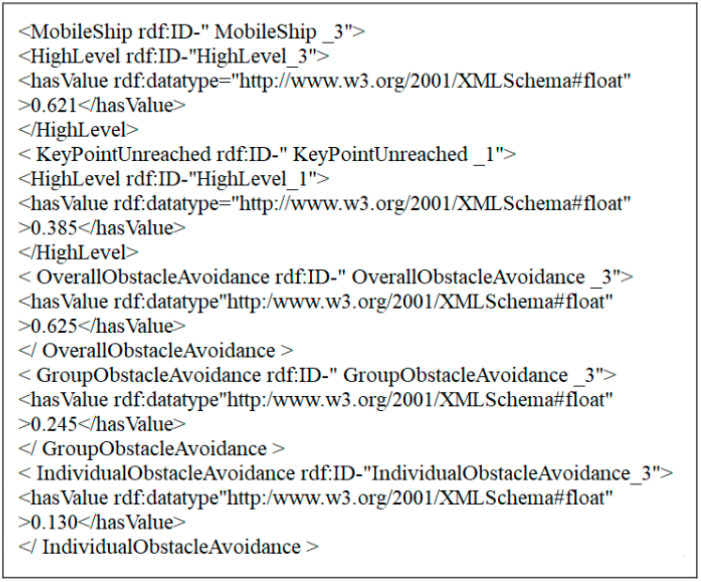
OWL format file.

When the system runs to 114 s, add a mobile ship, the probability of occurrence of the event of mobile ship is 0.358, the maximum probability of group collision avoidance is 0.422, then add another mobile ship, resulting in repeated attempts by MUSVs but difficult to reach the target. 116 s later the probability of occurrence of the key point unreachable event is 0.385, while the probability of occurrence of the event of mobile ship grows to 0.621, at this moment these two uncertain events overall collision avoidance probability 0.625 exceeds the trigger replanning threshold 0.6, at this time according to the underlying rules to determine the current uncertain events to meet the conditions for triggering replanning, the system calls the collision avoidance program, and then adjust the speed and direction of MUSVs in a timely manner. The probability of the occurrence of mobile ship emergence event and key point unreachable event gradually decreases until the overall collision avoidance probability, group collision avoidance probability and individual collision avoidance probability return to the initial value probability 0.3, 0.4 and 0.3 in 124 s. Figure 14 gives the contents of the corresponding OWL format files generated by the bottom layer when the moving ship emergence and critical point unreachability events occur in the experiment.

Where MobileShip _3 represents the generated instance of MobileShipAvoidance event, HighLevel_3 represents its event level, and 0.621 is its probability representation; KeyPointUnreached_1 represents the generated instance of KeyPointUnreached event, MediumLevel _1 represents its event level, and 0.385 is its GroupObstacleAvoidance _3 is the overall avoidance, 0.625 is the probability of the current overall avoidance. IndividualObstacleAvoidance _3 is the group avoidance, 0.245 is the probability of the current group avoidance. 3 individual avoidance, 0.130 indicates the probability of current individual avoidance.

## 6. Conclusions

In this paper, a cooperative perception framework for MUSVs with uncertain event detection, collision avoidance pattern recognition and environmental ontology model is proposed based on the consideration of various types of uncertainties occurring in the complex marine environment of MUSVs. By adopting ontology theory, an information representation model for cooperative perception of MUSVs is established so that the environmental information acquired by MUSVs cooperatively is shareable and reusable. Moreover, the combination of ontology and Bayesian networks is used to implement the process of uncertain event detection and collision avoidance pattern recognition. Simulation experiments are conducted in the context of the application of MUSVs performing formation safety navigation, and the experimental results mark that the uncertain event ontology and collision avoidance pattern ontology obtained by cooperative perception can provide effective data support for cooperative collision avoidance of MUSVs. This paper verifies the MUSVs cooperative perception method through the experiment. For the analysis of simulated online data, there is basically no misjudgment. Even if there is a possibility of misjudgment, the degree of threat of situation is very low, and the collision avoidance pattern has not changed and can be ignored. Therefore, the research of this paper has important theoretical significance and practical value.

## Figures and Tables

**Table 1:** Partial SWRL rules for detection of uncertain events of MUSVs.

Rule Name	Regular Expression
Rule 1	Even(?x) Λ Disto (?y) Λ hasDistance (?y,?z) Λ swrlb:lessThan(?z,70)→MobileShipAppears (?x)
Rule 2	Event(?x) Λ hasState(?x, ?y) Λ State(?y) Λ hasValue(?y, ?z) Λ swrlb:greaterThan(?z, 0)→KeyPointNotReachable (?x)
Rule 3	Event(?x) Λ hasDeviation(?x, ?y) Λ Deviation(?y) Λ hasDistance (?y, ?z) Λ swrlb:greaterThan(?z, 10)→OffCourse (?x)
Rule 4	Event(?x) Λ hasState(?x, ?y) Λ State(?y) Λ hasValue(?y, ?z) Λ swrlb:greaterThan(?z, 0y)→Deadlock(?x)
Rule 5	Event(?x) Λ Dense(?y) Λ hasCollisionRisk(?y, ?z) Λ swrlb:greaterThan(?z, 0.6)→DenseObstacles (?x)

**Table 2:** Conditional Probability Table.

	Dense Obstacles			Mobile Ship Appears			Off Course			Key Point Not Reachable			Deadlock	
	High	Medium	Low	High	Medium	Low	High	Medium	Low	High	Medium	Low	Yes	No
High	0.7	0.2	0.1	0.8	0.1	0.1	0.6	0.3	0.1	0.8	0.1	0.1	0.8	0.2
Medium	0.6	0.3	0.1	0.6	0.2	0.2	0.2	0.4	0.4	0.7	0.2	0.1	0.6	0.4
Low	0.1	0.3	0.6	0.1	0.2	0.7	0.1	0.3	0.6	0.2	0.2	0.6	0.2	0.8

**Table 3:** Cooperative perception output results.

Time (s)	Event Name	Event Probability	Overall Collision Avoidance Probability	Grouping Collision Avoidance Probability	Individual Collision Avoidance Probability
100	Mobile ship appeared event	0.723	0.116	0.653	0.231
101	Mobile ship appeared event	0.809	0.184	0.724	0.092
102	Mobile ship appeared event	0.728	0.119	0.643	0.238
103	Mobile ship appeared event	0.465	0.143	0.58	0.277
104	Mobile ship appeared event	0.448	0.133	0.3	0.567
105	Mobile ship appeared event	0.309	0.223	0.312	0.465
106	Mobile ship appeared event	0.191	0.296	0.313	0.391
107	Mobile ship appeared event	0.000	0.3	0.4	0.3

**Table 4:** Synergistic perception output results.

Time (s)	Event Name	Event Probability	Overall Collision Avoidance Probability	Grouping Collision Avoidance Probability	Individual Collision Avoidance Probability
114	Mobile ship appeared event	0.358	0.305	0.422	0.273
	Key point unreached event	0.000			
115	Mobile ship appeared event	0.557	0.462	0.458	0.358
	Key point unreached event	0.000			
116	Mobile ship appeared event	0.621	0.625	0.245	0.130
	Key point unreached event	0.385			
117	Mobile ship appeared event	0.693	0.763	0.163	0.074
	Key point unreached event	0.627			
118	Mobile ship appeared event	0.593	0.641	0.254	0.105
	Key point unreached event	0.521			
119	Mobile ship appeared event	0.358	0.445	0.372	0.183
	Key point unreached event	0.417			
124	Mobile ship appeared event	0.089	0.300	0.400	0.300
	Key point unreached event	0.124			

## Data Availability

Not applicable.
